# Empowerment as Mutual Aid

**DOI:** 10.1093/geroni/igaf122.1174

**Published:** 2025-12-31

**Authors:** Carole Cox

**Affiliations:** Fordham University, New York, New York, United States

## Abstract

Empowerment programs for grandparent caregivers have traditionally been conducted in-person, allowing participants to interact directly. However, COVID 19 pandemic made such gatherings impossible, where isolation was essential. Concomitantly, grandparents raising grandchildren faced heightened challenges, requiring assistance both for themselves and their caregiving roles. The goals of empowerment practice are to help clients achieve a sense of personal power, become more aware of connections between individual and community problems, develop skills, and be able to work collaboratively toward social change. A virtual empowerment group was developed to meet the needs of these families with the program under the auspices of the NY City Department for the Aging. The program consisted of 7 classes offered through Zoom. The only prerequisites to participating were to be a grandparent caregiver and have access to WIFI. The program graduated 95 grandparents, almost all were Black and low income living in New York public housing. Throughout the program, the participants in each cohort developed ties as they listened to and learned from each other. They exchanged phone numbers and emails and often contacted each other during the week. Given the isolation that many were experiencing, they benefited from a solid network as they interacted with others going through the same experiences. Even in normal times, raising a grandchild can be isolating as it can limit socialization. Consequently, virtual groups can be an important means to fostering ties and building social inclusion.

